# Analysis of serum and gene expression profile of cytokines (IL-6, TNF-α and TGF-β1) in chronic hepatitis C virus infection

**DOI:** 10.7717/peerj.13330

**Published:** 2022-04-20

**Authors:** Ismail Che Noh, Richard Avoi, Asma Abdullah Nurul, Imran Ahmad, Ruzilawati Abu Bakar

**Affiliations:** 1 Department of Biomedical Sciences, Faculty of Medicine and Health Sciences, Universiti Malaysia Sabah, Sabah, Malaysia; 2 Department of Pharmacology, School of Medical Sciences, Universiti Sains Malaysia, Kubang Kerian, Kelantan, Malaysia; 3 Department of Community and Family Medicine, Faculty of Medicine and Health Sciences, Universiti Malaysia Sabah, Sabah, Malaysia; 4 Biomedicine Programme, School of Health Sciences, Universiti Sains Malaysia, Kubang Kerian, Kelantan, Malaysia; 5 Department of Family Medicine, School of Medical Sciences, Universiti Sains Malaysia, Kubang Kerian, Kelantan, Malaysia

**Keywords:** Cytokine, Hepatitis C infection, Gene expression, Serum level

## Abstract

**Background:**

Chronic hepatitis C virus (HCV) infection is one of the major causes of liver cirrhosis and liver carcinoma. Studies have indicated that an imbalance of cytokine activities could contribute to the pathogenesis of chronic HCV infection. This study aimed to investigate serum levels and gene expression of cytokines (IL-6, TNF-α and TGF-β1) in chronic HCV infection among Malay male subjects.

**Methods:**

Thirty-nine subjects were enrolled from various health clinics in Kelantan, Malaysia, and divided into two groups: patients with chronic HCV infection (HP) and healthy control (HS). The serum cytokines IL-6, TNF-a—were measured using Luminex assay, and serum TGF-β1 was measured by ELISA. The mRNA gene expression for IL-6, TNF-α and TGF-β1 was measured by real-time reverse transcriptase polymerase chain reaction (RT-PCR).

**Results:**

There were statistically significant differences in the mean serum levels of IL-6, and TGF-β1 in HP compared to HS group (*p* = 0.0180 and *p* = 0.0005, respectively). There was no significant difference in the mean serum level of TNF-α in HP compared to HS group. The gene expression for the studied cytokines showed no significant differences in HP compared to HS group.

**Conclusion:**

Serum IL-6 was significantly associated with chronic HCV infection.

## Introduction

Hepatitis C virus (HCV) infection is identified as one of the major causes of chronic liver disease and is the leading indication for a liver transplant. It is estimated that about 58 million people are currently burdened by chronic HCV infection, with 1.5 million new infections recorded every year (World Health Organization, 2021). The incidence of HCV infection is higher in the eastern Mediterranean and Europe than in other regions. In Malaysia, the prevalence of HCV is about 2.5%, and the incidence of liver cirrhosis due to HCV is projected to increase steeply over the next two decades (McDonald et al., 2015).

Approximately 60–80% of acute HCV infections progress to chronic HCV infection, with symptoms presenting when liver damage progresses (Modi & Liang, 2008). Deaths occur due to complications when liver damage progresses to liver cirrhosis and carcinoma. The major risk factor for HCV transmission is intravenous drug use. About 60–80% of HCV cases in developed countries are caused by intravenous drug use (Dore et al., 2003). The risk of HCV infection increases with the duration of intravenous drug use (Latimer et al., 2009; Diaz et al., 2001). Sharing contaminated syringes or drug cookers and cotton exposes users to the bloodborne pathogen (Hagan et al., 2001). Other contributing factors include blood transfusions, nonsterile medical equipment, and unsafe medical or surgical-related procedures (Suan et al., 2019). The diagnosis of HCV infection involved screening test and confirmatory lab test. The screening of HCV infection based on serological assay to detect antibody from present or past infection and confirmed by molecular assay to detect HCV ribonucleic acid (RNA) (Clinical Practice Guidelines, 2019). The management of chronic HCV infection involved non-pharmacological and pharmacological intervention. The non-pharmacological approach such as behavioral modifications among active intravenous drug users is an integral part of the treatment. Patients on pharmacological treatment requires monitoring for adverse events and subjected to routine lab workout such as liver function test, serum creatinine and HCV RNA. The newer direct-acting antiviral drugs (DAAs) has better outcome, fewer side effects, and less monitoring required compared to standard treatment with pegylated interferon (World Health Organization, 2016).

Cytokines play a major role in modulating immune responses. Numerous studies have suggested that the progression of chronic hepatitis C lesions is associated with an imbalance of T helper (Th) 1 and 2—that is, upregulation of intrahepatic Th1 cytokines (*e.g*., interleukin-12, interleukin-18, tumor necrosis factor (TNF)-α and interferon (IFN)-γ) and as well as upregulation of Th2 cytokines (*e.g*., interleukin (IL)-4 and IL-10) (Fan et al., 1998). Th1 cells mainly secrete pro-inflammatory cytokines involved in cell-mediated immunity and play an important role in protecting against intracellular pathogens. By contrast, Th2 cells secrete anti-inflammatory cytokines and are involved in regulating the humoral immune response, with a high level of circulating cytokines indicating chronic infection (Bruno et al., 2011). However, previous studies have presented conflicting findings on the role of cytokines during HCV infection and the progression of the disease. Therefore, this study investigated the serum levels and gene expression of cytokines (IL-6, TNF-α, and tumor growth factor (TGF)-β1) in chronic HCV infection among Malay male subjects.

## Materials and Methods

### Study subjects

A total of 39 adult male subjects were enrolled in this study and recruited from various health clinics in the state of Kelantan, Malaysia, from July 2019 to December 2020. Study subjects were divided into two groups: 13 patients with chronic HCV infection (HP) and 26 control subjects (HS). The ethical clearances for the study were obtained from the Medical Research & Ethics Committee, Ministry of Health Malaysia (NMRR-19-399-45866), and USM Human Research Ethics Committee (USM/JEPeM/18010012), following the principles of Helsinki Declaration. Written informed consent was obtained from all study subjects. All participants were subjected to a medical history review, physical examination (weight, height, body mass index (BMI), and vital signs), and laboratory investigations (serum liver function test (LFT) and cytokine levels). Patients were diagnosed with chronic HCV infection when HCV antigen was present persistently for at least 6 months. All control subjects were screened for HCV infection using an SD Bioline HCV kit (Standard Diagnostic Inc., Suwon, South Korea). Any liver disease present caused by other etiological factors was excluded.

### Specimen collection and serum preparation

Blood samples were collected from each study subject by venipuncture under aseptic conditions. The blood samples were processed within 2 h of collection for peripheral blood mononuclear cells (PBMC) isolation and serum preparation. Six milliliters of the blood sample were used for the isolation of mononuclear cells by density gradient centrifugation using the Ficoll-Paque Gradient method. Another 4 ml of the whole blood samples were centrifuged at 1,000 × g for 15 min at room temperature for serum separation. The sera were aliquoted into 1.5 ml microcentrifuge tubes and stored in a freezer at −70 °C.

### Determination of serum cytokines

The serum levels of the studied cytokines for IL-6 and TNF-α was simultaneously determined using human premixed multi-analyte kits (catalog number LXSAHM) according to the manufacturer’s instructions on a Luminex 200 analyzer (R & D System, Minneapolis, MN, USA). The assay protocol was performed as follows: 50 μl of standard or sample was added to each well, followed by 50 μl of diluted microparticle cocktail. The mixture was incubated for 2 h at room temperature on a shaker at 800 rpm. Each well was then washed by removing the liquid, refilling it with 100 μl wash buffer, and removing the liquid again. This step was repeated three times. A total of 50 μl of a diluted biotin-antibody cocktail was then added to each well. The setup was covered and incubated for 1 h at room temperature on a shaker at 800 rpm. Each well was then washed three times with 100 μl wash buffer. Then, 50 μl of the diluted biotin-antibody cocktail was added to each well. The setup was covered and incubated for 1 h at room temperature on a shaker at 800 rpm. This was followed by a repeating washing step. Next, 50 μl of diluted streptavidin-PE was added to each well and incubated for 30 min at room temperature on the shaker at 800 rpm, followed by the washing step. The reaction analyses were performed within 90 min using a Luminex® 200. All samples were prepared in duplicate, and the average concentrations were measured.

The TGF-β1 concentration in the serum was determined using a Quantikine ELISA kit (R & D System, Minneapolis, MN, USA). A day before the measurement, the serum sample first was activated, by adding 20 μl of 1 N HCl to 40 μl serum sample. The sample then incubated at room temperatures for 10 min. The acidified sample was neutralized by adding 20 μl of 1.2 N NaOH/0.5 M HEPES and mixed well. Prior to the assay, the activated was diluted with calibrator diluent. The concentration read off the standard curve was multiplied by appropriate dilution factors. The measurement of TGF-β1 procedure involved by first pipetting the sample in a microplate precoated with a monoclonal antibody specific to TGF-β1. After washing away the unbound substance, the enzyme-linked polyclonal antibody specific for TGF-β1 was added to the wells. The wash step was repeated, and a substrate solution was added to develop the reaction color. The intensity of the color was measured, and the results reflected the proportion of TGF-β1 present in the samples.

### Evaluation of mRNA gene expression profile of cytokines

The mRNA gene expression was detected by real-time reverse transcriptase polymerase chain reaction (RT-PCR). The primer sequences are as listed in Table 1. The RNA samples were extracted from the isolated mononuclear cells using an RNeasy Mini Kit, with lot no 74104 according to manufacturer instructions. Spectrophotometry was used to determine the purity and concentration of RNA extracts. The quality of the RNA was reflected by a consistent ratio of 1.8 to 2.0. The extracted RNA was converted to cDNA using QuantiNova Reverse Transcription Kit, with lot no 205411 according to manufacturer instructions. The real-time RT-PCR was performed in a 20 μl reaction volume consisting of a mixture of 1 × SYBR Green PCR master mix, 1 × QN ROX reference dye, 0.7 μM forward primer, 0.7 μM reverse primer, and a total of 50 ng cDNA. The mixtures underwent initial heat activation at 95 °C for 2 min, 40 cycles of denaturation at 95 °C for 15 s, and combined annealing/extension at 60 °C for 30 s, followed by melting curve analysis. The analysis of relative gene expression analysis was performed using the 2^−ΔΔCT^ Method.

**Table 1:** Primer sequences for each cytokine and housekeeping gene.

Gene	Primer sequence
IL 6	Hs_IL6_1_SG QuantiTect Primer Assay
TNF-α	Hs_TNF_3_SG QuantiTect Primer Assay
TGF-β1	Forward: 5′ TGAACCGGCCTTTCCTGCTTC 3′ Reverse: 5′ GCGGAAGTCAATGTACAGCTG 3′
β-actin	Forward: 5′ TCTACAATGAGCTGCGTGTG 3′
(Housekeeping gene)	Reverse: 5′ GGTGAGGATCTTCATGAGGT 3′

### Statistical analysis

Statistical analysis was performed with Graph Pad Prism 9.0. The results are expressed as mean ± SD. The differences in the mean between the two groups were assessed by independent sample t-test or Mann-Whitney where appropriate. Correlation between serum cytokines and serum LFT, serum cytokines, and gene expression was analyzed and estimated using Pearson’s correlation (*r*). Results with *p* values ≤ 0.05 were considered statistically significant.

## Results

Table 2 shows the demographic background and serum LFT for study subjects. The demographic background data for study subjects were similar, except that the weight and BMI for healthy control subjects (HS) were higher compared to patients with chronic HCV infection (HS) group (*p* = 0.0133, *p* = 0.0101, respectively). For the liver profiles, two significant differences were observed in the mean concentration of albumin and the mean concentration of alkaline phosphatase (ALP). The mean level of albumin was higher, and the serum level of ALP was lower in the HP group compared to the HS group (*p* = 0.0149 and *p* = 0.0128, respectively). However, all the mean levels of the liver profiles were within the reference range.

**Table 2:** Demographic background and liver function test (LFT) result for all subjects.

	Patients with chronic HCV infection (HP) (*n* = 13) Mean (SD)	Control subjects (HS) (*n* = 26) Mean (SD)	Statistics *p*-value
Age (years)	42.9 (6.05)	38.4 (8.86)	0.1117
Height (m)	1.63 (0.58)	1.66 (0.09)	0.3975
Weight (kg)	60.69 (9.97)	72.38 (14.54)	0.0133*
BMI (kg/m2)	22.78 (3.44)	26.28 (3.96)	0.0101*
Brachial systolic BP (mm/Hg)	135.3 (23.21)	122.7 (9.32)	0.1555
Brachial diastolic BP (mm/Hg)	83 (10.21)	81.12 (9.89)	0.5823
Total protein (g/L)	82.46 (5.94)	77.65 (7.65)	0.0548
Albumin (g/L)	39 (5.58)	43.04 (4.13)	0.0149*
Total bilirubin (umol/L)	8.024 (7.69)	7.83 (3.59)	0.9130
Alanine aminotransferase, ALT (U/L)	47.54 (48)	40.65 (34.92)	0.6121
Aspartate transaminase, AST (U/L)	58.85 (38.52)	40.62 (29.52)	0.1093
Alkaline phosphatase, ALP (U/L)	112.8 (47.4)	79.81 (31.10)	0.0128*
Gamma-glutamyl transpeptidase, GGT (U/L)	87.54 (69.7)	56.92 (59.84)	0.1623

**Note:**

* Significant difference between the groups of HCV-infected patients (HP) and healthy controls (HS) with *p* < 0.05

**Figure 1:**
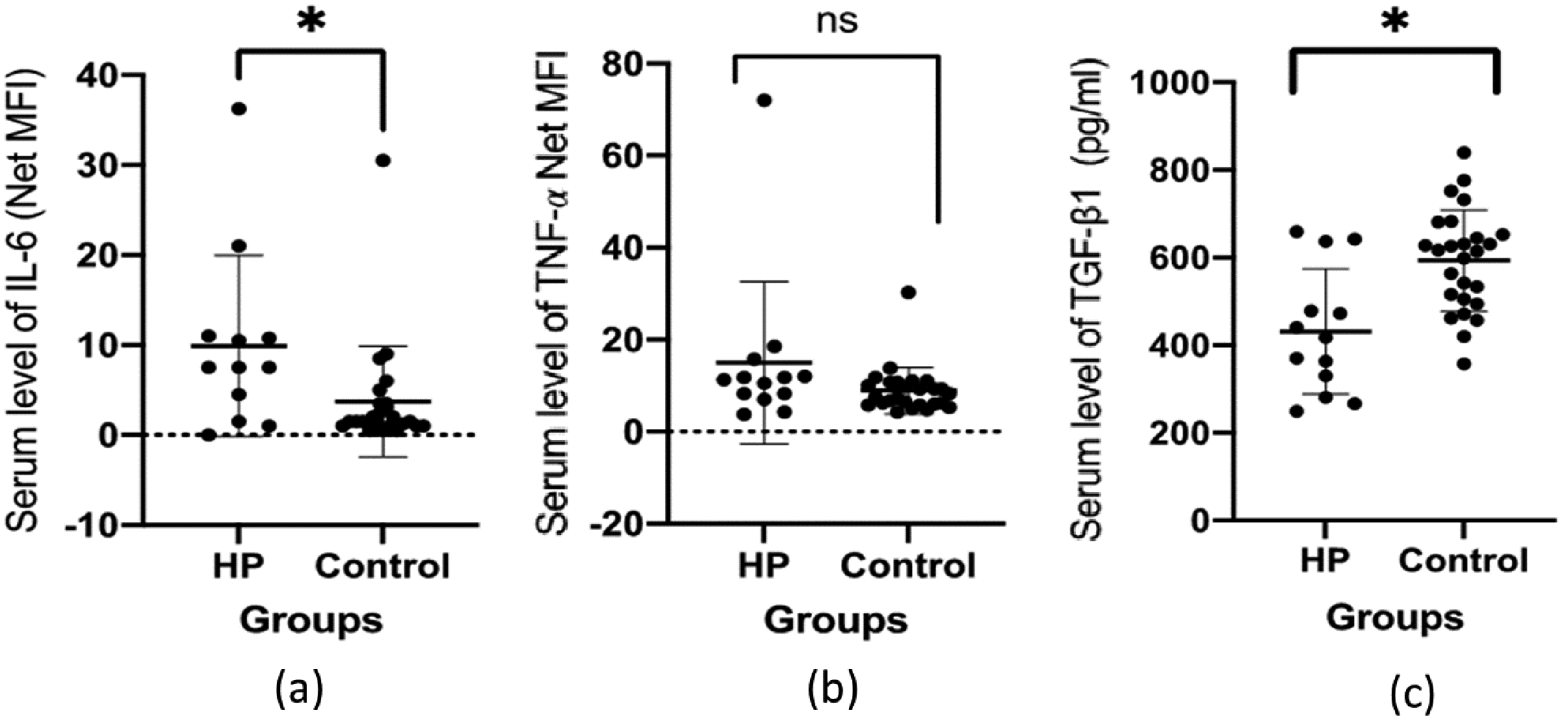
The pattern of the serum level of cytokines (A) IL-6 (B) TNF-a and (C) TGF-β1 among HCV-infected patients (HP) and healthy controls (HS). The asterisk (*) indicates significant difference between the groups of HCV-infected patients (HP) and healthy controls (HS) with *p* < 0.05.

Table 3 presents the mean serum levels of the studied cytokines among the two groups. The results for serum levels of IL-6, and TNF-α are reported in net MFI units, whereas serum levels for TGF-β1 are reported in the pg/ml unit. Statistically significant differences were observed in the mean serum levels of IL-6 and TGF-β1 among the groups. The mean serum level of IL-6 was higher in the HP group (9.92 ± 10.06), compared to the HS group (3.74 ± 6.17), with significant differences (*p* = 0.0180). The mean serum level of TGF-β1 was lower in the HP group (431.6 ± 142.7 pg/ml) compared to the HS group (593.6 ± 115.5) and the differences were statistically significant (*p* < 0.0001). The mean serum level of TNF-α was higher in the HP group (15 ± 17.62), compared to the HS group (8.93 ± 5.02). However, the differences were not statistically significant (*p* = 0.2016). Figure 1 summarizes the pattern of the mean serum levels for each studied cytokine.

**Table 3:** The mean serum levels of IL-6, TNF-a and TGF-β1 among study subjects.

	Patients with chronic HCV infection (HP) (*n* = 13) Mean (SD)	Control subjects (HS) (*n* = 26) Mean (SD)	Statistics *p*-value
Interleukin 6	9.92 (10.06)^a^	3.74 (6.17)^a^	0.0180*
TNF-α	15.00 (17.62)^a^	8.93 (5.02)^a^	0.0824
TGF-β1	431.6 (142.7)^b^	593.6 (115.5)^b^	0.0005*

**Notes:**

a Net MFI.

b pg/ml.

* Significant difference between the groups of HCV-infected patients (HP) and healthy controls (HS) with *p* < 0.05.

Table 4 presents the correlations between serum cytokines and serum LFT in the studied groups. In the HP group, the concentration of TNF-α has shown significant positive correlation with the serum level of ALP (*r* = 0.868, *p* = 0.0001) and gamma glutamyl transpeptidase (GGT) (*r* = 0.658, *p* = 0.014). The serum level of TGF-β1 also showed a significant positive correlation with serum GGT (*r* = 0.714, *p* = 0.006). In the HS group, there were significant negative correlations between the levels of IL-6 and total protein (*r* = −0.505, *p* = 0.0119) and albumin (*r* = −0.617, *p* = 0.0013). A similar pattern was observed between the levels of TNF-α and the total protein (*r* = −0.636, *p* = 0.0005) and albumin (*r* = −0.634, *p* = 0.0005) in this group. A significant negative correlation was also found between the serum levels of TNF-α and total bilirubin in HS group (*r* = 0.404, *p* = 0.0041). No other significant correlation was found in the other variables.

**Table 4:** Correlation between serum IL-6, TNF-a and TGF-β1 and serum LFT in HP and HS groups.

	IL-6		TNF-α		TGF-β1	
	*r*	*p*	*r*	*p*	*r*	*p*
*HP group*						
Total protein	0.144	0.654	0.234	0.441	0.0636	0.837
Albumin	0.230	0.450	0.319	0.288	0.159	0.605
Total bilirubin	−0.020	0.952	−0.287	0.342	−0.079	0.798
ALT	0.093	0.772	0.046	0.880	0.197	0.518
AST	0.347	0.270	>0.001	0.999	0.137	0.655
ALP	0.292	0.358	0.868	0.0001*	0.338	0.259
GGT	0.196	0.542	0.658	0.014*	0.714	0.006*
*HS group*						
Total protein	−0.505	0.0119*	−0.636	0.0005*	0.024	0.909
Albumin	−0.617	0.0013*	−0.634	0.0005*	0.317	0.115
Total bilirubin	−0.301	0.1523	−0.404	0.041*	0.361	0.070
ALT	−0.017	0.9377	−0.063	0.760	0.002	0.991
AST	−0.094	0.7716	−0.049	0.812	−0.156	0.447
ALP	−0.267	0.2071	−0.131	0.522	0.126	0.540
GGT	−0.114	0.5967	−0.148	0.470	0.173	0.399

**Note:**

* Statistically significant with *p* < 0.05.

Further analysis on the correlation serum-to-serum levels among the studied cytokines was made and the result presented in Table 5. There was a statistically significant negative correlation between the serum level of IL-6 and TNF-α in HP group (*r* = −0.908, *p* = 0.0001). Whereas the correlation between the serum level of IL-6 and TNF-α in the HS group was significantly positive (*r* = 0.859, *p* < 0.0001). There were no significant correlations observed between the serum level of TGF-β1 and serum IL-6 or TNF-α levels.

**Table 5:** Correlation on the serum-to-serum levels among studied cytokines in HP and HS group.

	HP group				HS group			
	TNF-α		TGF-β1		TNF-α		TGF-β1	
	*r*	*p*	*r*	*p*	*r*	*p*	*r*	*p*
IL-6	−0.908	0.0001*	−0.3099	0.328	0.859	<0.0001*	−0.328	0.118
TNF-α	–	–	−0.094	0.761	–	–	−0.1109	0.5896

**Note:**

* Significant difference between the groups of HCV-infected patients (HP) and healthy controls (HS) with *p* < 0.05.

Table 6 presents the mean mRNA gene expression for the studied cytokines. The mean gene expression for IL-6, TNF-α and TGF-β1 in the HP group were relatively lower than the HS group. However, all the differences were not statistically significant. The correlation between serum level and gene expression for studied cytokines was analyzed and the result presented in Table 7. There was a significant positive correlation on the gene expression with serum level of TNF-α in HS group (*r* = 0.657, *p* < 0.0443). However, no similar significant finding was made in the HP group. There were no significant correlations on the serum level with gene expression for IL-6 and TGF-β1 in both groups.

**Table 6:** The mean mRNA gene expression (2^−∆∆Ct^) of IL-6, TNF-α and TGF-β1 among study subjects.

	Patients with chronic HCV infection (HP) (*n* = 13) Mean (SD)	Control subjects (HS) (*n* = 26) Mean (SD)	Statistics *p*-value
Interleukin 6	16.26 (27.60)	50.06 (79.86)	0.5400
TNF-α	0.97 (1.66)	6.48 (8.66)	0.0817
TGF-β1	1.02 (0.55)	1.22 (1.22)	0.6719

**Table 7:** Correlation of serum level and gene expression profile of IL-6, TNF-a and TGF-β1 among study subjects.

Serum level/gene expression	Patients with chronic HCV infection (HP) (*n* = 13)		Control subjects (HS) (*n* = 26)	
	*r*	*p*	*r*	*p*
IL-6	−1.000	0.0833	0.055	0.9238
TNF-α	−0.400	0.7500	0.657	0.0443*
TGF-β1	0.281	0.7193	0.481	0.0840

**Note:**

* Significant difference between the groups of HCV-infected patients (HP) and healthy controls (HS) with *p* < 0.05.

## Discussion

Cytokines play a significant role in regulating the immune system and maintaining normal physiological processes. When there is a mismatch in the activities of pro-and anti-inflammatory components, cytokines contribute to disease progression and clinical outcomes. In this study, we evaluated the serum levels and gene expression of IL-6, TNF-α, and TGF-β1 in patients with chronic HCV infection. Further, this study analyzed the correlation between serum cytokines level and liver biomarkers, as well as between serum level and gene expression profile of the cytokines.

In response to tissue damage and infection, IL-6 is immediately and transiently produced by various cells, including fibroblasts, vascular endothelial cells, mast cells, macrophages, T cells, and B cells. IL-6 is a pleiotropic cytokine with both pro-and anti-inflammatory effects (Velazquez-Salinas et al., 2019). However, disruption in the production of IL-6 may lead to chronic inflammation and disease development (Reiser et al., 1991). Our findings suggest that IL-6 is associated with the development of chronic HCV infection. There were significant differences in the mean serum concentration of IL-6 among the studied subjects. The mean serum IL-6 was higher in patients with chronic HCV infection compared to healthy controls. The role of IL-6 in the pathogenesis of inflammatory diseases has been demonstrated in numerous human and animal model studies. In a study of two mouse models, IL-6 was shown to be required for the development of collagen-induced arthritis (El-Emshaty, Nasif & Mohamed, 2015). A study of 60 patients with chronic HCV infection indicated that the serum IL-6 concentration was increased by viral-induced inflammation prior to treatment (Malaguarnera et al., 1997).

TNF-α is an immune system modulator with a strong pro-inflammatory peripheral effect. It plays a crucial role in cell proliferation, differentiation, and apoptosis (Zelová & Hošek, 2013). TNF-α has been implicated in the pathogenesis of viral hepatitis and is associated with inflammation and fibrotic changes in hepatocytes (Moura et al., 2009). A study carried out on 53 patients with chronic HCV infection in Florida found significantly higher serum levels of TNF-α than those of healthy controls (Nelson et al., 1997). However, our study was unable to obtain a similar statistically significant result. In this study, we demonstrated that circulating TNF-α was not associated with HCV infection. The differences in the findings could be attributed to the sample size, study population, and ethnic background of the participants.

TGF-β1 regulates numerous cellular responses and the development and homeostasis of human tissues. During infection, TGF-β1 generally suppresses the inflammatory response. However, disruption in TGF-β1 activities has been implicated in the pathogenesis of various diseases (Kubiczkova et al., 2012). A study reported a significant association between serum TGF-β1 and chronic HCV infection in 75 patients compared to 15 healthy controls (Elbanan, Fathy & Ibrahim Hegazy, 2020). Another study of Egyptian patients indicated that TGF-β1 is associated with the severity of liver disease, in which patients with hepatocellular carcinoma had significantly higher serum TGF-β1 compared to patients with liver cirrhosis and healthy subjects (Kohla et al., 2017). However, a different finding was obtained in our study. The mean serum level of TGF-β1 was lower in HCV-infected patients, and the differences were statistically significant when compared to healthy controls. The different in the study design could lead to different finding. The negative association also can be contributed by the influence of the treatment. The mean TGF-β1 levels can significantly decrease at the end of anti-viral therapy and can persist to be lower than the baseline for at least 6 months (Kotsiri et al., 2016).

Our study did not establish association between gene expression profile of studied cytokines with chronic HCV infection. Overall, the gene expression for IL-6, TNF-α and TGF-β1 were relatively lower in patients with chronic HCV infection compared to healthy subjects but, the differences were not statistically significant. Further analysis on correlation of gene expression with serum level of the cytokines in chronic HCV infection revealed no significant findings except for TNF-α. The serum level of TNF-α was moderately, positively correlated with the gene expression profile in patients with chronic HCV infection. Here, there were inconsistency in the correlation between the mRNA gene expression profiles and protein levels. A study has shown that there was positive correlation between mRNA gene expression and protein level across 14 types of human tissues (Kosti et al., 2016). However, an earlier study done concluded that the correlations between mRNA and protein expression levels were varied across different genes and mRNA expressions not necessarily always predictive towards protein levels (Guo et al., 2008).

A few notable findings were made from analysis on the correlation of the serum level of cytokines with serum LFT. The serum level of TNF-α was significantly correlated with serum ALP and GGT in chronic HCV patients. A similar observation was made between the serum levels of TGF-β1 and GGT. ALP is mainly released from the liver and bone. A moderate elevation of serum ALP can be non-specific. A markedly elevated serum ALP is clinically significant, as it may indicate liver disease (Lowe, Sanvictores & John, 2021). GGT is mostly secreted by hepatocytes. It is a routine assayed blood analyte for liver disease, especially alcohol-induced liver injury (Koenig & Seneff, 2015). An activation of TNF-α system and elevated serum ALP and GGT levels could reflects active liver damages.

It was found that in this study the serum TNF-α and IL-6 levels were inversely correlated with total protein and albumin levels in healthy subjects. In liver disease, total protein and albumin levels can be decreased. The synthesis of albumin by the liver can be inhibited by TNF-α and IL-6 (Gounden, Vashivit & Jialal, 2021). A significant elevation of serum TNF-α and IL-6, but decreased protein or albumin levels, are suggestive for extensive liver damage.

In general, liver biomarkers may appear to be normal in early phase HCV infection. But when the disease progresses and becomes more severe, liver enzymes may be elevated. As we found in our study, the serum levels of IL-6, and TGF-β1 were significantly higher in patients with chronic HCV infection, and mostly independent with the levels of liver enzymes. This finding suggests that IL-6 and TGF-β1 system are activated in chronic HCV infection and are sensitive markers to the inflammatory changes occurs in the liver.

The small cohort in our study may significantly limit the clinical interpretation of our findings. However, this study provides a good basis for further clinical investigations of potential biomarkers relevant to chronic HCV infection. Future studies should involve a larger cohort and could be expanded to include an investigation of the potential association between cytokines and the severity of liver disease and progress, as well as responses to treatment.

## Conclusions

Our study suggests that serum levels of IL-6 is associated with chronic HCV infection. However, serum level of TGF-β1 was negatively associated with chronic HCV infection and there was no significant association observed for TNF-α. The same result was found with gene expression profiles of the cytokines. We did not find it to be associated with chronic HCV infection.

## Supplemental Information

10.7717/peerj.13330/supp-1 Supplemental Information 1 Luminex Analysis.

10.7717/peerj.13330/supp-2 Supplemental Information 2 qRTPCR.

10.7717/peerj.13330/supp-3 Supplemental Information 3 ELISA.

## Additional Information and Declarations

### Data Availability

The following information was supplied regarding data availability: The raw measurements are available in the Supplemental Files.
